# Initial Outcomes of Freehand Transperineal Biopsies Regarding Diagnostic Value and Safety: An Early Experience at King Fahad Specialist Hospital, Buraydah, Saudi Arabia

**DOI:** 10.7759/cureus.39318

**Published:** 2023-05-21

**Authors:** Hatim Alnosayan, Mohannad A Alharbi, Adel H Alharbi, Abdullah S Aloraini, Abdulhamid M Alfayyadh, Mohammed Almansour

**Affiliations:** 1 Department of Urology, College of Medicine, Qassim University, Qassim, SAU; 2 Department of Urology, King Fahad Specialist Hospital, Qassim, SAU

**Keywords:** regional anesthesia, diagnostic efficacy, complications, transperineal biopsy, prostate cancer

## Abstract

Background: Prostate cancer is a common type of cancer in Saudi Arabia with a high incidence rate. Trans-rectal ultrasound guided prostatic biopsy (TRUSBx) has been the standard diagnostic study for prostate cancer since a landmark study in 1989 which showed that it is better than digitally directed biopsy sampling of the prostate. As an alternative to TRUSBx, transperineal biopsies (TPBx) have gained popularity as they give a higher accuracy rate and avoid many complications. A new study has been conducted in Riyadh, Saudi Arabia to compare TRUSBx and TPBx showed that TPBx has a significantly higher detection rate of prostate cancer cases compared to TRUSBx (45.1% vs. 29.1%, *p*=0.003).

The aim of this study is to determine the diagnostic value and safety of freehand transperineal prostate biopsy in patients with an elevated prostatic specific antigen (PSA) and/or abnormal digital rectal exam in King Fahad Specialist Hospital KFSH in Buraydah, Qassim region, Saudi Arabia.

Methods: This is an observational retrospective study of all patients (n=39) who underwent transperineal biopsies at KFSH to assess the diagnostic value and safety of the procedure.

Results: The mean age of the patients was 70.3 (SD 10.1) years. The most commonly found diagnosis was adenocarcinoma (61.5%), and incidence of complications was detected in (5.1%) of the patients.

Conclusion: We concluded that the freehand technique TPBx has a high accuracy rate in detecting prostatic cancer. However, the learning curve could be a limiting factor in implementing it. Increasing the number of biopsies could positively affect diagnostic accuracy, especially with our low complication rate in this procedure. A low number of biopsies in the older age group can give an accurate result with a low risk of complications. Although template-guided TPBx and robot-guided TPBx are better options, the freehand technique represents a cost-effective and time-saving alternative. However, more studies are needed to compare the outcome of such a technique.

## Introduction

In Saudi Arabia, prostate cancer is a prevalent malignancy with a high incidence rate (7.0 per 100.000) [1]. Its frequency has considerably increased within the Saudi population during the last three decades. Prostate cancer incidence climbed eight times from 150 to 1300 cases between 1990 and 2016, according to a 2018 study [2]. Since a seminal study in 1989 that demonstrated its superiority over digitally directed prostate biopsy samples, trans-rectal ultrasonography-guided prostatic biopsy (TRUSBx) has become the accepted method for diagnosing prostate cancer [3].

A TRUSBx is carried out when a patient's test results show a high prostate-specific antigen (PSA) level or an abnormal digital rectal exam [4]. However, compared to the final Gleason score at radical prostatectomy, TRUSBx has a false negative rate of 15%-46% and a tumour undergrading rate of up to 38% since needle positioning about tumour location is essentially random [5]. The needle will contaminate the prostate with bacterial flora from the rectum during a TRUSBx because it will pass through the rectal wall. Targeted biopsies can reduce this danger, although saturation biopsies may need the needle to pass more than 20 times. Each time, rectal bacteria spread to the blood and prostate. Consequently, it is essential to use prophylactic antibiotics [6,7]. According to some research, the prevalence of febrile infections after TRUS-guided biopsy samples has increased over the past 15 years and now affects about 4% of patients, with up to 3.1% needing hospitalization [8,9]. Fluoroquinolone resistance has probably contributed to the rise in infection rates [10].

Transperineal biopsies (TPBx), an alternative to TRUSBx, have grown in favour since they do not have these complications. Under local or regional anaesthesia, TPBx is performed, and the needle is inserted into the clean perineal skin [11,12]. It leads to a significantly lower incidence of infection-related consequences, with sepsis rates close to zero [13,14]. TPBx has some obvious drawbacks, including a longer duration than TRUSBx and a higher rate of post-procedure pain complaints [15,16]. Although numerous studies were conducted comparing the TPBx and TRUSBx techniques' detection rates, the conclusions of the two approaches' respective detection rates were controversial [17-19].

A recent study comparing TRUSBx and TPBx in Riyadh, Saudi Arabia, found that TPBx had a considerably greater prostate cancer detection rate than TRUSBx (45.1 % vs 29.1 %, p=0.003). The same study compared the problems between the two techniques, finding that hematuria and urinary retention were the most often found issues in both groups, with no discernible difference between them. The clear distinction was that 3.6% of patients reported rectal bleeding following trans-rectal biopsy [15]. Based on the experience of King Fahad Specialist Hospital in Buraydah, Qassim region, Saudi Arabia, this study aims to determine the initial outcomes of freehand TPBx in terms of diagnostic value and safety.

## Materials and methods

This observational retrospective study was conducted at King Fahad specialist hospital in Buraydah, Saudi Arabia, to all patients who underwent TPBx from April 2020 to August 2022. All the patients in this research have met the inclusion criteria by having an elevated PSA (≥ 4 ng/mL) and /or abnormal digital rectal exam (suspicious nodule, hard nodular prostate, lobar asymmetry, the presence of indurations and obliteration of the median groove). The procedure was done in freehand technique TPBx under regional anaesthesia. Patients with a different diagnostic biopsy procedure or anaesthesia method have been excluded. In our sample, we measured age, BMI, patient’s main complaint, comorbidities, initial PSA level, prostate volume, PSA density, imaging that had been done to the patient before the procedure, prostate imaging-reporting and data system (PIRADS) for patients who did MRI, procedure time, number of biopsies, diagnosis of the biopsies, length of hospital stay and the complications.

Statistical analysis

Descriptive statistics were calculated and summarized as numbers, percentages, and mean. The relationship between the age group and length of stay according to the baseline characteristics of the patients was performed using Fischer Exact test and independent sample t-test. The normality test was performed using the Shapiro-Wilk test. A P-value of 0.05 was considered statistically significant. The data were analyzed using Statistical Packages for Social Sciences (SPSS) version 26 (IBM Corp., Armonk, NY).

## Results

We reviewed 39 patients who underwent TPBx. As seen in Table 1, the mean age of the patients was 70.3 (SD +/- 10.1) years. The most commonly found diagnosis was adenocarcinoma (61.5%), while the most frequently used imaging method was MRI (41%). Patients with 5/5 PIRADS constitute 38.5%. Incidence of complications was detected in two patients (5.1%), one patient had retrograde ejaculation, and the other patient complained of haematuria. Mortality rates happened in two patients (5.1%) after six months of follow-up with the patients. Furthermore, the mean BMI of the patients was 24.9 (SD 4.66) kg/m^2^. The mean initial PSA (ng/ml), prostate volume (ml), and PSA density (ng/ml2) were 36.6, 73.5, and 0.49, respectively. The mean number of biopsies taken from the patients was 11.3. The mean Gleason scores for patients diagnosed with adenocarcinoma were 7.84, while the mean procedure time (minutes) and length of the hospital (days) were 70.9 and 2.69, respectively.

**Table 1:** Baseline characteristics of the patients who underwent transperineal prostatic biopsy (n=39) BPH: benign prostatic hyperplasia. CT: computed tomography. MRI: magnetic resonance imaging. US: ultrasound. BMI: body mass index. PSA: prostate specific antigen. SD: standard deviation. PIRADS: prostate imaging-reporting and data system.

Study variables	N (%)
Diagnosis	
Adenocarcinoma	24 (61.5%)
BPH	12 (30.8%)
BPH with prostatitis	01 (02.6%)
Carcinoma in situ	01 (02.6%)
Non-conclusive	01 (02.6%)
Prior test	
CT	04 (10.3%)
MRI	16 (41.0%)
US	03 (07.7%)
MRI + CT	02 (05.1%)
US + CT	03 (07.7%)
US + MRI	11 (28.2%)
PIRADS	
3/5	05 (12.8%)
4/5	09 (23.1%)
5/5	15 (38.5%)
Unknown	10 (25.6%)
Complication	
None	37 (94.9%)
Retrograde ejaculation	01 (02.6%)
Hematuria	01 (02.6%)
Mortality	
Yes	02 (05.1%)
No	37 (94.9%)
	Mean ± SD
Age in years	70.3 ± 10.1
BMI (kg/m^2^)	24.9 ± 4.66
Initial PSA (ng/ml)	36.6 ± 39.0
Prostate volume (ml)	73.5 ± 47.5
PSA density (ng/ml^2^)	0.49 ± 0.61
Number of biopsies	11.3 ± 7.69
Gleason score	7.84 ± 1.07
Procedure time (minutes)	70.9 ± 25.5
Length of hospital stay in days	2.69 ± 1.61

**Figure 1:**
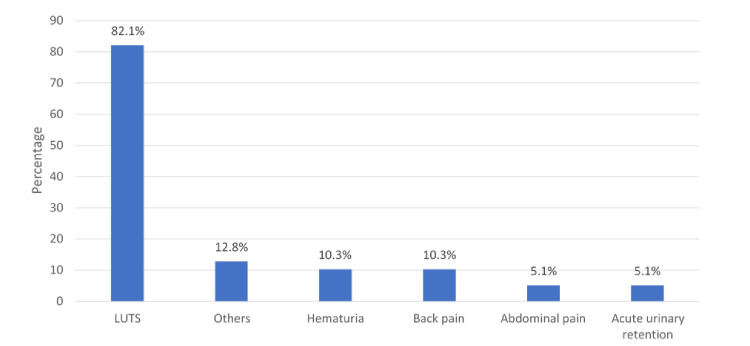
Primary complaint

In Figure 1, the most prominent complaint of the patients was lower urinary tract symptoms LUTS (82.1%), followed by haematuria (10.3%) and back pain (10.3%).

**Figure 2:**
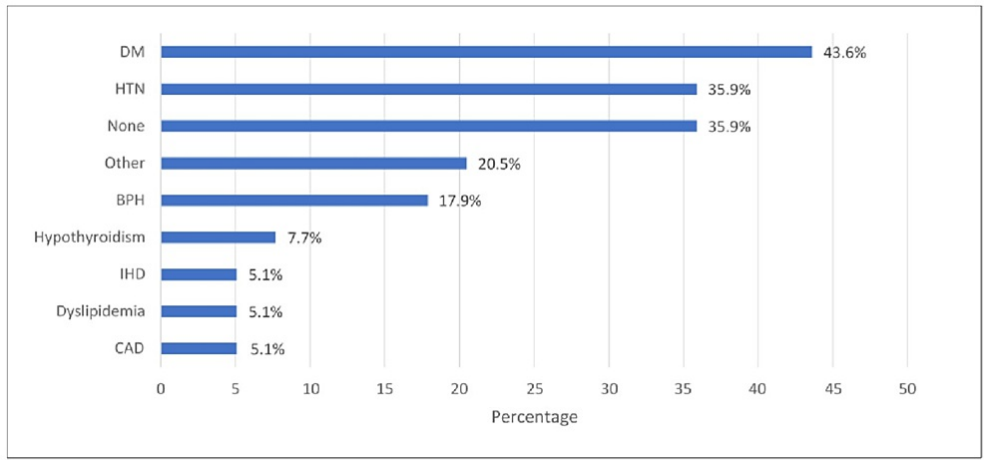
Associated comorbidities

In Figure 2, the most commonly known associated comorbidity was DM (43.6%), followed by hypertension (35.9%) and benign prostatic hyperplasia (BPH) (17.9%).

When measuring the safety and efficacy of TPBx in relation to the age group in Table 2, it was found that the prevalence of back pain (p=0.047) was statistically significantly higher in the older age group (age >70 years). We also observed that a higher BMI level (p=0.049) and a higher number of biopsies (p=0.030) were more associated with the younger age group (age ≤70 years). In comparison, a higher value of initial PSA (p=0.005) was more associated with the older age group (age >70 years). However, the differences in diagnosis, PIRADS, related comorbidities, prostate volume, PSA density, Gleason score, procedure time, and length of hospital stay between the age groups were not significantly different (p>0.05).

**Table 2:** Relationship between the age group and the baseline characteristics of the patients (n=39) * Some patients have multiple complaints. § P-value has been calculated using Fischer Exact test. ‡ P-value has been calculated using an independent sample t-test. ** Significant at p<0.05 level.

Factor	Age group		P-value ^§^
	Age ≤70 years N (%) ^(n=20)^	Age >70 years N (%) ^(n=19)^	
Diagnosis			
Adenocarcinoma	09 (50.0%)	15 (78.9%)	0.091
BPH	09 (50.0%)	04 (21.1%)	
PIRADS			
3/5	04 (23.5%)	01 (08.3%)	0.469
4/5	04 (23.5%)	05 (41.7%)	
5/5	09 (52.9%)	06 (50.0%)	
Complaint *			
LUTS	18 (90.0%)	14 (73.7%)	0.235
Hematuria	03 (15.0%)	01 (05.3%)	0.605
Abdominal pain	02 (10.0%)	0	0.487
Back pain	0	04 (21.1%)	0.047 **
Urinary retention	01 (05.0%)	01 (05.3%)	1.000
Others	02 (10.0%)	03 (15.8%)	0.661
Associated comorbidities			
Yes	13 (65.0%)	12 (63.2%)	1.000
No	07 (35.0%)	07 (36.8%)	
	Mean ± SD	Mean ± SD	P-value ^‡^
BMI (kg/m^2^)	27.4 ± 4.74	23.2 ± 3.54	0.049 **
Initial PSA (ng/ml)	20.1 ± 22.8	54.1 ± 45.3	0.005 **
Prostate volume (ml)	80.4 ± 53.7	64.8 ± 38.3	0.336
PSA density (ng/ml^2^)	0.35 ± 0.48	0.69 ± 0.73	0.102
Number of biopsies	13.8 ± 7.99	8.58 ± 6.51	0.030 **
Gleason score	7.90 ± 1.10	7.80 ± 1.08	0.824
Procedure time (minutes)	69.0 ± 24.5	72.9 ± 27.1	0.640
Length of hospital stay in days	2.45 ± 0.69	2.95 ± 2.19	0.341

In Table 3, it was observed that there were no significant differences in all of the tested variables, whether it is a short duration hospital stay (≤2 days) or a more extended hospital stay (>2 days) (all p>0.05).

**Table 3:** Relationship between the LOS and the baseline characteristics of the patients (n=39) * Some patients have multiple complaints. § P-value has been calculated using Fischer Exact test. ‡ P-value has been calculated using an independent sample t-test. ** Significant at p<0.05 level. LOS: Length of stay

Factor	Length of hospital stay		P-value ^§^
	≤2 days N (%) ^(n=22)^	>2 days N (%) ^(n=17)^	
Diagnosis			
Adenocarcinoma	12 (54.5%)	12 (80.0%)	0.165
BPH	10 (45.5%)	03 (20.0%)	
PIRADS			
3/5	03 (16.7%)	02 (18.2%)	1.000
4/5	06 (33.3%)	03 (27.3%)	
5/5	09 (50.0%)	06 (54.5%)	
Complaint *			
LUTS	18 (81.8%)	14 (82.4%)	1.000
Hematuria	03 (13.6%)	01 (05.9%)	0.618
Abdominal pain	0	02 (11.8%)	0.184
Back pain	01 (04.5%)	03 (17.6%)	0.300
Urinary retention	01 (04.5%)	01 (05.9%)	1.000
Others	03 (13.6%)	02 (11.8%)	1.000
Associated comorbidities			
Yes	13 (59.1%)	12 (70.6%)	0.518
No	09 (40.9%)	05 (29.4%)	
	Mean ± SD	Mean ± SD	P-value ^‡^
Age in years	69.9 ± 8.84	71.1 ± 11.7	0.655
BMI (kg/m^2^)	24.9 ± 4.15	25.0 ± 5.31	0.982
Initial PSA (ng/ml)	28.9 ± 38.8	46.7 ± 38.1	0.161
Prostate volume (ml)	71.5 ± 39.9	76.3 ± 57.8	0.771
PSA density (ng/ml^2^)	0.43 ± 0.66	0.59 ± 0.55	0.451
Number of biopsies	10.9 ± 6.65	11.8 ± 9.05	0.704
Gleason score	7.58 ± 1.08	8.08 ± 1.04	0.257
Procedure time (minutes)	68.4 ± 24.9	74.1 ± 26.8	0.496

## Discussion

Few investigations on TPBx have been conducted since (1989) by Hodge et al., who first described the systematic biopsy protocol [3]. Some studies showed no significant differences between transperineal biopsies and trans-rectal biopsies regarding diagnostic accuracy [18,20-22]. Other studies have compared the complication rates between TPBx and TRUSBx and demonstrated that sepsis was higher in the TRUSBx technique, while acute urinary retention was higher in the TPBx approach [22-25].

In our study, there were no significant differences in the safety and efficacy of TPBx with the age group. Moreover, we have observed that there were no significant differences in all of the tested variables, whether it is a short duration hospital stay (≤2 days) or a more extended hospital stay (>2 days) (all p>0.05).

In our sample, prostate volume was 73.5 ml (SD 47.5) which is comparable and in the range of other reports [15,20,21]. The mean PSA was 36.6 ng/ml (SD 39), which is higher than other similar studies like Takenaka et al. [21], Cerruto et al. [20], Rabah et al. [15], with their PSA results being (17.1±30.1), (15.95±41.04), (14.2±5) ng/ml, respectively. This high mean PSA might be attributed to the delayed presentation due to the lack of information and utilization of screening tests in our community, as suggested by Arafa and Rabah [26]. Furthermore, a higher value of initial PSA (p=0.005) was more associated with the older age group (age >70 years). About histopathology, 24 (61.5%) biopsies were confirmed to be adenocarcinoma, 13 (33.4%) came out as BPH and one patient had carcinoma in situ while one biopsy was inconclusive. Our cancer detection rate is comparable with the cancer detection rate in a study by Takenaka et al., which is 39.7% [21], reflecting the efficacy and reliability of such a technique. Additionally, we have compared the PIRAD scores with the cancer detection rate and found that 86.7% of patients with a PIRAD score of 5/5 and 44.4% with a PIRAD of 4/5 had cancer.

A systematic review summarized more than 29 studies for freehand TPBx under local anaesthesia and showed an average procedure time of 13.1 minutes [27]. However, our study's mean procedure time was 71 (SD 25.5) minutes. This time might be attributed to the senior surgeon's involvement and training of other surgeons and the learning curve of such a procedure.

The mean number of core biopsies done to our patients was 11.3 (SD 7.69) biopsy (maximum 29, minimum 2). A higher number of biopsies, 13.8 (SD 7.99) (p=0.030), were associated with the younger age group (age ≤70 years) of the patients. Low PIRADS and less incidence of metastasis at presentation for this age group could explain the high number of biopsies needed. Also, this higher number can be beneficial since reports have demonstrated the safety of increasing the number of biopsies with no significantly different complication rate [21,28].

It was observed that there were no significant differences in complication rate, whether it is a short duration hospital stay (≤2 days) or a more extended hospital stay (>2 days) (all p>0.05). However, the cost of the stay was not calculated. Although the procedure is being done under regional anaesthesia, our patients had a mean length of hospital stay of 2.69 (SD 1.61) days (maximum 8 days, minimum 0 days), and this time was primarily preoperative since our patients needed extensive evaluation and workup.

The complication rate was 5.1% of those who did TPBx, with only one patient who had retrograde ejaculation and the other having hematuria. These results are similar to Rabah et al. (6.3%), Di Franco et al. (4%), and Halpern et al. (5.4%) reports, with hematuria and urinary retention being the most common complications in all of them [15,18,25]. However, with Huang et al. complication rate reached 15.3% [22]. Notably, no infection was documented with all of our patients who underwent TPBx, and it has not been reported as a complication of such a technique by others.

In comparison, template-guided TPBx on real-time ultrasound (US) imaging is the most common technique clinicians use that allows for standardized sampling and is more accessible for less-experienced clinicians. However, using a brachytherapy grid and associated equipment is more time-consuming and expensive than free-hand TPBx [29]. Also, robot-guided TPBx uses robotic guidance for the US probe and needle placement for prostate biopsy. Robot-guided TPBx has been shown to detect better clinically significant prostate tumours with fewer cores extracted and to have more accurate needle placement than template-guided TPBx. However, many urologists may not readily have access to this technology because it is expensive [30].

## Conclusions

We concluded that the freehand technique TPBx has a high accuracy rate in detecting prostatic cancer. However, the learning curve could be a limiting factor in implementing it. Increasing the number of biopsies could positively affect diagnostic accuracy, especially with our low complication rate in this procedure. A low number of biopsies in the older age group can give an accurate result with a low risk of complications. Although template-guided TPBx and robot-guided TPBx are better options, the freehand technique represents a cost-effective and time-saving alternative. However, more studies are needed to compare the outcome of such a technique.
